# Impact of Atypical Antipsychotics on Sexual Dysfunction

**DOI:** 10.1192/j.eurpsy.2026.12116

**Published:** 2026-07-31

**Authors:** S. Aouadi, A. Aissa, Y. Ben Othman, A. Hlaoui, R. Hosni, R. Jomli

**Affiliations:** 1 Avicenne Psychiatry; 2 Psychiatry, Hospital Razi, Tunis, Tunisia

## Abstract

**Introduction:**

Atypical antipsychotics (AAPs) are commonly prescribed in psychiatry. However, their effects on sexual function remain underestimated, despite significantly impacting quality of life and treatment adherence.

**Objectives:**

To evaluate the prevalence of sexual dysfunction (SD) induced by AAPs in stabilized patients and to identify associated factors.

**Methods:**

A cross-sectional observational study was conducted in the Avicenne Psychiatry Department at the El Razi Psychiatric Hospital in Tunis between January and April 2025. Thirty-six stabilized outpatients treated with AAPs were included. Data were collected using a pre-established form and two assessment tools: the Psychotropic-Related Sexual Dysfunction Questionnaire (SALSEX) and the Arizona Sexual Experience Scale (ASEX). Statistical analysis was performed using IBM SPSS 25.

**Results:**

The mean age of the patients was 42 years (range: 28-66), with a male predominance (70.6%). 54% had not exceeded secondary school level, and 58% were unemployed with a low socioeconomic status. Regarding lifestyle habits, 94.1% of patients were smokers, and 94.1% consumed alcohol and cannabis. Clinically, 29.4% of patients were diagnosed with type 1 bipolar disorder, 29.4% with schizophrenia, 17.6% with type 2 bipolar disorder, and 21.6% with schizoaffective disorder.

The prevalence of sexual dysfunction was 42%, distributed as 31% moderate and 15% severe. Among the affected patients, 47% were in a relationship, of whom 16% reported marital conflicts. However, no significant correlation was found between marital conflicts and SD (p = 0.569). Furthermore, 47% had no sexual education, while 53% had a “taboo” based education, with no significant link to SD (p = 0.226).

Analysis of treatments revealed that 26% of patients were on clozapine, 26% on risperidone, 47% on olanzapine, and 10% on amisulpride. A significant association was observed between SD and risperidone (p = 0.041) and a trend towards significance with amisulpride (p = 0.067). In contrast, no significant correlation was found with the other AAPs.

**Conclusions:**

Atypical antipsychotics present a differential risk for SD, with a more pronounced impact observed with risperidone and amisulpride. Systematic evaluation of these side effects is essential to optimize treatment adherence and patient quality of life.

**Disclosure of Interest:**

None Declared

